# Post-Stroke Dysphagia: Moving Toward Evidence-Based, Recovery-Oriented Systems of Care

**DOI:** 10.1007/s11910-026-01507-0

**Published:** 2026-07-24

**Authors:** Heather Shaw Bonilha, Brittany N Krekeler, Janina Wilmskoetter, Sandeep Kumar, Erin L. Reedy, Wuwei Feng, Salman Ikramuddin, Fadi Nahab

**Affiliations:** 1 Department of Communication Sciences and Disorders, University of South Carolina, Columbia, SC USA; 2 Department of Otolaryngology-Head and Neck Surgery, University of Cincinnati College of Medicine, Cincinnati, OH USA; 3 Department of Rehabilitation Sciences, Medical University of South Carolina, Charleston, SC USA; 4 Harvard Medical School Beth Israel Deaconess Medical Center, Boston, MA USA; 5 Department of Otolaryngology - Head & Neck Surgery, Department of Speech Pathology, University of Rochester Medical Center, Rochester, NY USA; 6 Division of Stroke & Vascular Neurology, Duke Comprehensive Stroke Center Director, Neuromodulation & Stroke Recovery Lab, Department of Neurology, Duke University School of Medicine, Durham, NC USA; 7 Department of Neurology, Division of Stroke and Vascular Neurology, Duke University School of Medicine, Durham, NC USA; 8 Departments of Neurology and Pediatrics, Division of Vascular Neurology, Emory University Emory Healthcare, Atlanta, GA USA

**Keywords:** Dysphagia, Stroke, Videofluoroscopy, Gastrostomy tube, Neuroplasticity, Rehabilitation

## Abstract

**Purpose of review:**

This review examines challenges in post-stroke dysphagia management, including access to swallowing imaging, enteral feeding decisions, and barriers to reassessment and rehabilitation. It evaluates how healthcare systems and practice patterns influence swallowing outcomes and stroke recovery.

**Recent Findings:**

Recent literature emphasizes that post-stroke dysphagia is a dynamic condition requiring longitudinal management rather than a temporary acute complication. Evidence supports validated dysphagia screening followed by clinical swallowing evaluation and swallowing imaging for accurate diagnosis and treatment planning. Studies highlight variability in gastrostomy tube practices, post-acute access to swallowing imaging, continuity of care, and the psychosocial burden of oral restriction and feeding decisions after stroke.

**Summary:**

Dysphagia management systems remain fragmented, contributing to inconsistent diagnosis, reassessment, access to intervention, and prolonged unnecessary oral intake restrictions. Future efforts should prioritize rehabilitation pathways that support recovery-oriented dysphagia management.

## Introduction

Post-stroke dysphagia remains a persistent challenge for stroke systems of care because swallowing recovery often extends beyond the healthcare settings in which key management decisions are made. Although swallowing impairments are common after stroke and strongly influence medical complications, rehabilitation participation, and quality of life [1, 2], dysphagia management often remains fragmented across acute and post-acute care settings. Decision making in acute post-stroke dysphagia management is time-sensitive and constrained by hospital- and systems-level barriers, particularly related to when and how to assess swallowing impairments, the need for enteral feeding, discharge planning, and access to rehabilitation services. At the same time, growing evidence highlights the importance of recovery-oriented dysphagia care that extends well beyond the acute hospitalization period [3–5].

Traditionally, dysphagia management after stroke has focused primarily on reducing aspiration risk and preventing pneumonia during hospitalization. However, contemporary evidence increasingly supports conceptualizing post-stroke dysphagia as a dynamic rehabilitation condition requiring longitudinal reassessment and coordinated management across the recovery continuum [3, 6–8]. The 2026 American Heart Association/American Stroke Association (AHA/ASA) acute ischemic stroke guideline continues to emphasize early dysphagia screening prior to oral intake and highlights the importance of multidisciplinary stroke systems that support timely swallowing evaluation and management [9], alongside 2016 AHA/ASA guidance related to rehabilitation and recovery [10]. Similarly, the European Stroke Organisation (ESO) and European Society for Swallowing Disorders (ESSD) guidelines emphasized the need for structured dysphagia pathways that include acute clinical screening and assessment using swallowing imaging, and rehabilitation throughout stroke recovery [2]. Although these and other guidelines emphasize rehabilitation across the recovery continuum, they provide limited guidance regarding optimal timing of reassessment and ongoing post-acute swallowing rehabilitation [3]. Likewise, recent reviews continue to characterize post-stroke dysphagia as an evolving neurophysiologic disorder requiring repeated assessment and individualized rehabilitation beyond acute hospitalization [1, 2, 11].

Despite broad recognition of these principles, clinical practice often remains misaligned with the available evidence. Data continue to demonstrate substantial variation in dysphagia screening and assessment, access to swallowing imaging, timing and determination for long-term access to alternative means of nutrition (e.g., gastrostomy tube [G-tube] placement), reassessment pathways, and rehabilitation services across healthcare systems. Published evidence, including observational studies, randomized trials, and meta-analyses, supports the use of validated dysphagia screening within hyperacute stroke pathways to reduce pneumonia and other adverse outcomes [12–15]. However, screening alone does not characterize the physiologic mechanisms underlying swallowing impairment. These features can only be definitively evaluated with swallowing imaging when concern persists after clinical swallowing evaluation (CSE) [1, 2]. Dysphagia outcomes are influenced not only by patient-specific clinical factors, but also by use of non-validated dysphagia screens, variable access to swallowing imaging, inconsistent diagnostic coding, communication challenges across care settings, involvement of patients and care partners in decision making, and systems-level pressures related to enteral nutrition and discharge planning. Decisions made during this brief period frequently influence rehabilitation access, long-term swallowing outcomes, overall health, and quality of life long after hospital discharge.

Post-stroke dysphagia is an underrecognized but significant determinant of stroke recovery. Within this context, this review examines post-stroke dysphagia through a recovery-oriented systems-of-care framework, with emphasis on how clinical pathways and healthcare delivery influence swallowing outcomes across the stroke continuum of care. Specifically, the review discusses the role of swallowing imaging as a foundational component of stroke care, evaluates current practices surrounding decision-making for long-term nutritional access (e.g., G-tube), and highlights barriers to reassessment, rehabilitation, and continuity of care after hospital discharge. By integrating emerging evidence with systems-level considerations, this review identifies opportunities to move dysphagia management beyond acute risk mitigation toward coordinated, longitudinal models that better support stroke recovery and quality of life.

## Post-Stroke Dysphagia: An Underrecognized Determinant of Stroke Recovery

The clinical consequences of post-stroke dysphagia are significant and multifaceted. Dysphagia is strongly associated with aspiration pneumonia, malnutrition, dehydration, prolonged hospitalization, institutionalization, and increased mortality risk [16–20]. Pneumonia is associated with the greatest excess mortality among post-stroke medical complications and occurs disproportionately among patients with dysphagia, who experience an 8- to 10-fold greater risk than stroke survivors without dysphagia [19–21].

In addition to pulmonary complications, inadequate nutrition and hydration resulting from dysphagia negatively affect overall health, participation in rehabilitation, and neurologic recovery [22–25]. Although malnutrition and dehydration are widely recognized complications, nutrition remains underrepresented within the dysphagia literature relative to aspiration and pneumonia. Nutritional deficits may emerge rapidly during acute hospitalization and persist throughout recovery, particularly among patients with prolonged restrictions in oral intake. Recent reviews highlight the need for greater integration of nutrition assessment and management within interdisciplinary stroke rehabilitation models, recognizing nutrition as both a consequence of dysphagia and a potentially modifiable determinant of recovery [22–25].

The impact of post-stroke dysphagia extends beyond medical complications to include substantial reductions in quality of life [26]. Eating and drinking are central to daily function, personal autonomy, and social participation. Consequently, dysphagia frequently contributes to social withdrawal, anxiety, caregiver burden, and reduced participation in meaningful activities [26–32]. These psychosocial consequences may also contribute to depression, which is common after stroke. Emerging evidence suggests that swallowing impairment may increase the risk and severity of depressive symptoms through its effects on social engagement, independence, and quality of life [33, 34]. Post-stroke dysphagia should therefore be understood not simply as a swallowing impairment, but as a multidimensional condition with physiologic, functional, emotional, and social consequences.

Post-stroke dysphagia is one of the most common complications of acute stroke, yet it remains substantially underrecognized within contemporary stroke systems of care. Prospective swallowing imaging-based studies suggest that as many as 80% of patients experience swallowing impairment during acute hospitalization, rates far exceeding those documented in administrative records or routine clinical coding [16, 35, 36]. In a review of AHA Get with the Guidelines-Stroke (GWTG-Stroke) data from 2010 to 2019, dysphagia screening was reported for only 70% of patients during acute hospitalization for acute ischemic stroke or intracerebral hemorrhage (*n* = 2,749,288), with 17.2% failing the screen and 53.4% passing [37]. Comparing these registry data with prospective studies reveals two persistent problems: approximately 30% of patients are not receiving dysphagia screens, or those screens are not being documented, and a significant proportion of those who are screened receive false-negative results, likely due to use of non-validated screening tools or inconsistent implementation of validated screening protocols [12]. This discrepancy highlights a persistent diagnostic gap with important implications for patient outcomes and healthcare systems. Recent retrospective and registry-based studies continue to demonstrate substantial variability in dysphagia identification and documentation across stroke systems, suggesting that many patients with clinically significant swallowing impairments remain unrecognized during acute hospitalization [16, 36, 38].

Importantly, swallowing recovery after stroke is a dynamic process. Although recovery is most rapid during the first one to three months after stroke, it commonly continues throughout the first year and may occur even years later through neuroplastic adaptation and targeted rehabilitation [39–45]. This trajectory mirrors recovery patterns observed in other post-stroke impairments and supports the role of task-specific, physiologically informed intervention [42–45]. Although acute hospitalization represents only a brief portion of the recovery timeline, typically lasting about one week [46], critical decisions regarding oral intake restrictions, enteral nutrition, intervention approaches, discharge disposition, and follow-up care are often made before long-term swallowing prognosis can be confidently determined [47, 48].

Systems-level constraints further compound these challenges. Acute care clinicians often operate within fragmented transition-of-care systems where post-discharge rehabilitation, follow-up, and information sharing may be limited [49–51]. As a result, many stroke survivors are ultimately lost to follow-up after discharge [52]. This limited longitudinal visibility reduces opportunities to evaluate how acute care practices influence downstream outcomes such as pneumonia, readmissions, nutritional status, and functional recovery [3, 47, 50], and may contribute to the persistence of practices that are not aligned with recovery-oriented dysphagia management. As a result, the downstream consequences of acute dysphagia management decisions, including prolonged oral intake restrictions, unnecessary long-term enteral nutrition, or missed opportunities for rehabilitation and re-evaluation, often remain invisible to the clinicians in the acute care setting who made the initial recommendations. Emerging literature highlights substantial gaps in long-term dysphagia management guidance, post-acute reassessment infrastructure, and continuity across rehabilitation transitions [53–56], contributing to inconsistent follow-up and reduced access to recovery-oriented swallowing rehabilitation.

## Swallowing Imaging as a Foundational Component of Acute Stroke Care

Given the substantial clinical consequences of post-stroke dysphagia and the dynamic nature of swallowing recovery, early and accurate characterization of physiologic swallowing impairment is critical. However, dysphagia screenings and CSEs alone cannot reliably identify the physiologic mechanisms underlying dysphagia that result in aspiration and pharyngeal residue. Accurate diagnosis and treatment planning therefore require swallowing imaging, now recognized as a foundational component of contemporary dysphagia diagnosis and management and increasingly recognized as a foundational element of post-stroke care.

Within contemporary stroke systems, the pathway for identifying patients with post-stroke dysphagia begins with a validated dysphagia screen, often completed by nursing due to their 24-hour staffing. Current quality initiatives support dysphagia screening before any oral intake, including medications, based on evidence linking screening protocols to reduced rates of post-stroke pneumonia and other adverse outcomes [12–15, 57, 58]. Dysphagia screening using validated assessments, of which several are available, is therefore a critical component of acute stroke care and often serves as the gateway through which patients gain access to further swallowing evaluation and rehabilitation services [59–64].

The second step is typically a CSE, also termed a “bedside” swallowing evaluation, conducted by a speech-language pathologist (SLP). While the CSE remains an important component of evidence-based dysphagia care, it does not permit direct visualization of swallowing physiology, airway protection, or pharyngeal residue accumulation. Consequently, a CSE alone is often insufficient for definitive dysphagia diagnosis in patients with acute stroke [65–69]. This limitation is particularly important because clinically significant swallowing impairments frequently occur without obvious outward signs or symptoms. Among post-stroke patients who aspirate, approximately 70% do so silently, without cough, throat clearing, wet vocal quality, or other observable indicators of airway invasion [70]. Cognitive impairment, sensory and/or motor deficits, fatigue, communication disorders, and impaired self-awareness may further complicate detection of swallowing impairment after stroke [71, 72]. That is, many patients may not recognize that they have dysphagia or ongoing aspiration owing to the covert nature of post-stroke swallowing impairments [73–75]. As a result, patient report and observation alone may substantially underestimate swallowing impairment in some individuals. Conversely, other patients may demonstrate behaviors suggestive of aspiration despite no airway invasion events when examined with swallowing imaging. Consequently, clinically significant dysphagia may be missed in some patients and overestimated in others. This dissociation between clinical presentation and physiologic impairment represents a fundamental challenge in post-stroke dysphagia management and reinforces the need for and critical role of swallowing imaging after the CSE.

While the CSE identifies clinical signs and symptoms of dysphagia, it cannot determine aspiration with certainty or provide the detailed physiologic information regarding laryngeal, pharyngeal, and upper esophageal mechanisms underlying identified swallowing impairments; information is critical for fully assessing risk and developing targeted treatments. In this regard, CSEs should be viewed similarly to neurologic examinations. A neurologist would not rely solely on a bedside neurologic examination to define infarct location, hemorrhage, or vascular status. Rather, the clinical examination identifies risk and informs subsequent diagnostic testing. Likewise, CSEs provide important clinical information but cannot reliably characterize swallowing physiology. Recent state-of-the-science reviews and the European Stroke Organization (ESO) and European Society of Swallowing Disorders (ESSD) guidelines similarly emphasize that swallowing imaging remains essential for accurate physiologic characterization, and recommend its use when clinical findings are inconclusive, when silent aspiration is suspected, or when physiologic characterization is needed to guide management [1, 2].

Aside from identifying deficits, swallowing imaging is critical for determining the therapeutic approach. Swallowing imaging serves not merely to confirm dysphagia, but as the primary means of identifying the physiologic abnormalities that inform prognosis, rehabilitation targets, oral intake recommendations, and compensatory strategies. This distinction is clinically important because commonly used management approaches, including liquid viscosity modifications and compensatory postures, may improve swallowing safety in some patients while proving ineffective or even detrimental in others, underscoring the need for physiologic assessment to guide individualized recommendations.

The two principal swallowing imaging modalities, videofluoroscopic swallow study (VFSS, also termed Modified Barium Swallow Study [MBSS]) and flexible endoscopic evaluation of swallowing (FEES), provide distinct but complementary information. Both modalities permit identification of aspiration and pharyngeal residue. VFSS provides a comprehensive physiologic assessment across the oral and pharyngeal phases of swallowing and allows characterization of timing, coordination, structural movement, and bolus flow patterns that cannot be visualized via FEES. In contrast, FEES offers advantages related to bedside accessibility, secretion management assessment, and visualization of tissue integrity and vocal fold function not visible on VFSS [76]. Recent comparative reviews emphasize that VFSS and FEES should not be conceptualized as competing examinations, but rather as discrete and equally essential procedures that address different clinical questions [1, 76, 77]. Consequently, the question is often not whether VFSS or FEES should be performed, but which examination is most appropriate at a given point in the patient’s recovery trajectory. FEES is particularly valuable for medically unstable patients and serial clinical reassessment, whereas VFSS provides more comprehensive physiologic characterization and may be an ideal imaging study to prioritize as patients stabilize and approach discharge. Many patients may have indications for both examinations at different points during their hospitalization and recovery.

Despite the critical role of swallowing imaging in diagnosis and treatment planning, it is often treated as discretionary rather than a foundational diagnostic procedure. This approach differs markedly from many other areas of acute care, where imaging of internal structures and physiologic processes is considered an expected component of clinical decision making. Yet major swallowing management decisions are often made without direct visualization of the physiologic events themselves. In many hospitals, these decisions must be based solely on the clinical hypothesis derived from the CSE because the availability of swallowing imaging does not match clinical demand. These constraints are often related to equipment availability, staffing limitations, procedural prioritization, and reimbursement-related challenges [78, 79].

The importance of swallowing imaging during acute hospitalization is further amplified by substantially reduced access to imaging in many post-acute settings. Skilled nursing facilities and subacute rehabilitation settings frequently face transportation barriers, equipment limitations, staffing constraints, fragmented referral pathways, and restrictive reimbursement structures that limit access to VFSS and FEES [48, 54, 80–84]. Consequently, swallowing recommendations established during acute hospitalization may persist long after they are clinically necessary. For many stroke survivors, acute hospitalization represents the most accessible, and in some cases, the only realistic opportunity to characterize swallowing physiology. Acute hospitalization, therefore, serves as a critical window in which swallowing physiology can be accurately characterized, rehabilitation targets established, and recovery-oriented management initiated.

The consequences of limited access to swallowing imaging extend beyond diagnostic uncertainty. In post-acute settings, reliance solely on CSE may result in both under-recognition of clinically significant dysphagia and unnecessary restriction of oral intake in patients whose swallowing physiology has not been re-evaluated with imaging. Despite neurologic recovery, swallowing recommendations established during acute hospitalization are often carried forward across care settings. Consequently, many patients continue oral intake restrictions or enteral nutrition support despite substantial improvements in swallowing function due to a lack of access to swallowing imaging [81, 85].

Ultimately, swallowing imaging occupies a central position within the post-stroke dysphagia care pathway. Imaging findings influence oral intake recommendations, rehabilitation planning, prognostic deliberations, decisions regarding enteral nutrition, and longitudinal reassessment strategies. Improving access to clinically indicated swallowing imaging during acute stroke hospitalization, therefore, represents an important opportunity to strengthen recovery-oriented dysphagia care across the continuum of stroke recovery. Table 1 summarizes best practice recommendations for acute care post-stroke dysphagia.

## Enteral Nutrition Decisions and the Challenge of Early Prognostication

Accurate characterization of swallowing impairment is particularly important because it informs one of the most consequential decisions made during acute stroke hospitalization: how nutrition, hydration, and medication administration will be managed during recovery. Dysphagia frequently limits safe oral intake during the early post-stroke period, yet prolonged inadequate nutrition and hydration are associated with worse functional outcomes, increased morbidity, and increased mortality [22–25]. Medication administration may also depend on enteral access, creating additional pressure to establish reliable feeding routes early.

Because G-tubes can theoretically be removed if swallowing function recovers, placement is often viewed as a relatively low-risk or reversible intervention during periods of prognostic uncertainty. However, the practical consequences of G-tube placement frequently extend well beyond the acute hospitalization period. Once placed, G-tubes may persist because of limited reassessment, barriers to repeat swallowing imaging, and challenges accessing rehabilitation services. As a result, decisions initially intended to support short-term recovery may ultimately influence long-term swallowing management and quality of life.

Decisions regarding enteral nutrition can be complicated by the highly dynamic nature of swallowing recovery after stroke. Meaningful improvement may occur within days or weeks, with continued recovery extending months beyond hospitalization [39–45]. As a result, clinicians are often asked to make long-term nutritional management decisions during a period of substantial physiologic uncertainty, before the patient’s ultimate swallowing trajectory can be confidently determined. Although several prognostic tools, such as the Predictive Swallowing Score (PRESS), have been developed to support feeding tube decision-making, none have been validated using swallowing imaging, and the variables included across models vary considerably [86–89]. Accurately predicting long-term enteral nutrition, therefore, remains difficult.

Current guidelines generally support G-tube placement only when prolonged inability to swallow safely is anticipated [56], with existing recommendations favoring nasogastric (NG) tube feeding during the initial weeks after stroke before considering long-term enteral access [9, 90]. However, real-world practice frequently diverges from these recommendations. Acute care length of stay has progressively shortened, often resulting in discharge timelines that precede recommended waiting periods for G-tube decisions [46–48]. Acute care clinicians are, therefore, frequently asked to determine long-term enteral nutrition needs before the trajectory of swallowing recovery becomes apparent.

Systems-level pressures further complicate these decisions. Many post-acute facilities remain reluctant to accept patients with NG tubes because of staffing concerns, radiographic confirmation requirements, reimbursement limitations, and care coordination challenges, should G-tube placement ultimately become necessary [3, 47, 48, 50]. Consequently, acute care hospitals often assume responsibility for determining whether patients should undergo G-tube placement prior to discharge. In practice, this means that decisions regarding enteral nutrition are often driven not only by swallowing prognosis and supplemental nutritional needs, but also by discharge logistics and post-acute placement barriers.

One potential strategy for reducing this prognostic pressure would be greater acceptance of patients with NG tubes across post-acute care settings [91, 92]. Although such an approach would require infrastructure and workflow changes, it could allow additional time for swallowing recovery and reassessment while maintaining closer alignment with existing guideline recommendations regarding the timing of G-tube decisions. Importantly, this approach would also create opportunities for repeat swallowing imaging and more individualized prognostication before committing patients to long-term nutrition access.

The consequences of these decisions often extend far beyond the acute hospitalization period. Longitudinal outcome studies demonstrate that many stroke survivors experience substantial swallowing recovery after discharge, including recovery sufficient to permit feeding tube removal [53, 86, 93]. Even when enteral nutrition is medically necessary during acute recovery, feeding tubes are placed without structured reassessment plans. In effect, interventions intended to be provisional too often become permanent because healthcare systems do not prioritize ongoing swallow evaluation and recovery-oriented follow-up [54–56]. Contemporary neuroplasticity-oriented dysphagia rehabilitation literature emphasizes the importance of active swallowing, task-specific practice, and physiologically targeted intervention during recovery [40, 42]. Excessive reliance on strict non-oral nutrition without structured swallowing rehabilitation that includes therapeutic oral intake may therefore contribute to persistent swallowing impairment through mechanisms of disuse [94, 95]. While enteral nutrition may be medically necessary during acute recovery, ongoing reassessment and active rehabilitation remain essential to maximizing recovery potential. Studies examining post-acute populations demonstrate that many patients with oral intake restrictions, including many with G-tubes, no longer demonstrate clinically significant dysphagia when reassessed with swallowing imaging [81, 85, 96], suggesting that inadequate reassessment contributes to unnecessary long-term restrictions. These outcomes are often invisible to acute care clinicians, who rarely receive longitudinal feedback regarding swallowing recovery or long-term feeding tube status after discharge.

Another important consideration is that feeding tubes do not eliminate aspiration risk. Patients discharged with G-tubes continue to experience substantial rates of aspiration pneumonia and hospital readmission [55]. These observations reinforce that enteral nutrition decisions are risk-benefit assessments rather than definitive solutions to aspiration risk. The relationship between dysphagia and pneumonia is complex and influenced by factors beyond aspiration alone, including oral health and hygiene, airway protection, foregut function, immune status, medical comorbidity, and overall functional status. Oral intake restriction may be appropriate when swallowing imaging demonstrates unsafe physiology; however, it should not be viewed as a default or permanent intervention without ongoing reassessment and rehabilitation [68]. Clear documentation regarding swallowing prognosis, rehabilitation goals, and plans for reassessment should accompany enteral nutrition decisions, and repeat swallowing imaging should be incorporated into post-discharge care pathways whenever possible.

The complexity of enteral nutrition decisions extends beyond medical risk alone. Eating and drinking carry profound personal, social, and cultural significance for many patients. Stroke survivors frequently describe loss of eating and drinking as one of the most psychologically devastating consequences of their stroke [97, 98]. Dysphagia may be experienced not only as a loss of nutritional independence, but also as social isolation, loss of pleasure, and erosion of personal identity. Consequently, nutritional decisions should not be viewed solely through the lens of aspiration prevention, but also through their effects on autonomy, participation, and quality of life.

Recognizing the central role of eating and drinking in quality of life requires clinicians to engage patient autonomy as a core component of oral intake and nutrition decisions. In a prospective study of hospitalized stroke patients, two-thirds reported that long-term tube dependence on tube feeding would be equivalent to or worse than death [99]. Ethical obligations in dysphagia management, therefore, extend beyond risk reduction and include supporting informed patient decision-making when preferences, quality of life, and medical recommendations intersect.

For patients with severe dysphagia, poor overall prognosis, substantial comorbidity, or limited likelihood of meaningful recovery, comfort feeding (i.e., eating and drinking with an informed consent of risk or ‘feeding at risk’) represents an ethically grounded and clinically acceptable option that prioritizes dignity and patient-defined quality of life. Structured comfort-feeding pathways in non-stroke populations have been shown to be feasible and acceptable, with improvements in care processes and staff–patient communication [100]. Whether similar pathways improve patient-centered outcomes in severe post-stroke dysphagia warrants further investigation. Nevertheless, comfort-feeding discussions should be recognized as an important component of patient-centered dysphagia care and shared decision-making after stroke.

## From Acute Diagnosis to Longitudinal Recovery: Improving Continuity, Reassessment, and Rehabilitation Access After Stroke

Although critical decisions regarding swallowing management are often made during acute hospitalization, recovery from post-stroke dysphagia extends far beyond the inpatient setting. Increasingly, the literature identifies systems-level gaps in continuity of care, communication, reassessment, and rehabilitation access as major contributors to long-term dysphagia morbidity after stroke [53–56]. Contemporary guidelines similarly emphasize the need for coordinated interdisciplinary communication and patient and care partner education; however, they provide no guidance regarding optimal timing for reassessment or ongoing post-acute swallowing rehabilitation [2, 56, 101, 102]. Given the influence of acute hospitalization on long-term swallowing outcomes, discharge planning should extend beyond immediate safety concerns and include explicit consideration of recovery potential, rehabilitation goals, and plans for longitudinal follow-up.

For many stroke survivors, discharge from acute hospitalization marks the beginning of an inconsistent and fragmented process for ongoing assessment and management of swallowing impairment. The quality and continuity of dysphagia care often varies considerably across inpatient rehabilitation facilities, skilled nursing facilities, outpatient services, and home-based care. Each of these settings operates within different resource constraints and may have variable access to clinicians with expertise in dysphagia assessment, swallowing imaging, and rehabilitation. As a result, opportunities for recovery may depend as much on post-acute service access rather as on swallowing impairment severity.

Access to swallowing imaging after acute discharge remains particularly variable. Patients receiving care in settings with established dysphagia care pathways and ready access to VFSS or FEES may be better positioned to undergo timely reassessment than those in settings where imaging is difficult to obtain because of transportation barriers, scheduling delays, and care coordination challenges. Yet reassessment, with swallowing imaging, should not be viewed as an optional component of care. Because swallowing function frequently changes during recovery, repeat swallowing imaging is often necessary to evaluate recovery, refine rehabilitation goals, guide oral intake advancement, and determine whether enteral nutrition remains necessary. Regardless of discharge disposition, reassessment should be recognized as an expected component of recovery-oriented dysphagia management rather than a service limited to higher resourced environments.

Communication across care settings represents another major challenge. Information transfer between acute care hospitals, rehabilitation facilities, outpatient providers, home health teams, patients, and care partners is frequently incomplete or inconsistent [3, 50, 103]. As a result, swallowing recommendations may be continued without understanding the physiologic rationale underlying them. Patients may undergo redundant evaluations, receive conflicting recommendations, or remain on unnecessarily restricted oral intake because updated swallowing information is unavailable. These inconsistencies may also erode trust among patients and care partners who receive differing recommendations without clear explanations.

Improving continuity will require more standardized communication regarding swallowing assessment findings and rehabilitation plans. Discharge documentation should clearly describe swallowing physiology, the rationale underlying oral intake recommendations, compensatory strategies that were trialed and their outcomes, anticipated recovery potential, and the recommended timing for reassessment. Whenever possible, swallowing imaging reports and access to recorded examinations should accompany patients during transitions between care settings. Such information may reduce redundant testing, improve continuity of care, and facilitate more individualized and effective rehabilitation planning.

Patients and care partners likewise require greater support in navigating post-stroke dysphagia recovery. Many families remain unaware that swallowing recovery may continue long after discharge or require repeat evaluation [103]. For patients discharged with G-tubes, additional burdens often fall upon patients, families, and care partners who must manage enteral feeding equipment despite having no prior experience. Acute care teams can help address these challenges through structured education and anticipatory guidance emphasizing the dynamic nature of swallowing recovery, the potential need for reassessment, and the importance of ongoing rehabilitation participation.

Access to skilled dysphagia rehabilitation represents another important systems-level challenge. Health system structures, reimbursement policies, and regulatory requirements substantially influence access to both swallowing rehabilitation and repeat swallowing imaging across post-acute care settings [80–84]. In many healthcare systems, documentation generated during acute hospitalization serves as the foundation for establishing medical necessity and authorizing services. Swallowing imaging findings, dysphagia severity classifications, oral intake recommendations, and documentation of functional limitations may therefore determine whether patients receive swallowing rehabilitation and reassessment. Failure to accurately identify and characterize swallowing impairment may limit access to recovery-oriented services long after the patient leaves the hospital. Greater alignment between reimbursement structures, quality oversight mechanisms, and evidence-based dysphagia management practices may help reduce disparities in access to recovery-oriented care. Tables 1 and 2 summarize best practice recommendations for acute care and post-acute care post-stroke dysphagia.

**Table 1:** Acute care post-stroke dysphagia best practice recommendations

Domain	Best practice recommendation
Dysphagia screening	Administer a validated nurse-led swallowing screen to all (suspected) stroke patients prior to any oral intake, including medications; document screening results and ensure screening is completed regardless of stroke severity or clinical presentation.
Clinical swallowing evaluation	Refer all patients who fail dysphagia screening for timely speech-language pathology evaluation; treat clinical swallowing evaluation findings as a guide to diagnostic testing rather than a definitive characterization of swallowing physiology.
Swallowing imaging	Obtain swallowing imaging (VFSS or FEES) when dysphagia screening is failed, when findings from clinical swallowing evaluation are inconclusive, when silent aspiration is suspected, or when physiologic characterization is needed to guide intervention and oral intake decisions; treat imaging as a foundational diagnostic procedure rather than a discretionary resource.
Imaging modality selection	Apply FEES for medically unstable patients requiring assessment on the ward or evaluation of secretion management and laryngeal integrity; apply VFSS for comprehensive physiologic characterization of oral and pharyngeal swallowing mechanisms; recognize that both modalities may be clinically indicated during hospitalization.
Oral hygiene	Integrate oral hygiene into routine acute care plans to reduce aspiration pneumonia risk; recognize that oral health is a modifiable determinant of respiratory outcomes.
Nutrition integration	Incorporate systematic nutrition assessment into acute dysphagia management; diagnose and monitor malnutrition and dehydration; engage dietitians as members of the interdisciplinary rehabilitation team.
Enteral feeding decisions	Base enteral feeding decisions on swallowing imaging findings whenever possible; follow current guideline recommendations favoring nasogastric tube feeding during initial weeks after stroke before transitioning to long-term enteral access; avoid premature gastrostomy tube placement when meaningful swallowing recovery remains possible.
Patient and care partner education	Provide structured education regarding the nature of dysphagia, the rationale for oral intake recommendations, the dynamic course of swallowing recovery, and the importance of ongoing reassessment and rehabilitation participation.
Shared decision-making	Incorporate patient preferences and quality of life goals into feeding and management decisions; discuss comfort feeding (eating and drinking with acknowledged risk) as an ethically appropriate option for patients with severe dysphagia, poor overall prognosis, or patient-defined priorities that do not align with strict aspiration precautions.
Discharge documentation	Provide explicit discharge documentation describing swallowing physiology, rationale for oral intake recommendations, compensatory strategies trialed and their outcomes, anticipated recovery potential, and recommended timing and setting for reassessment; accompany patients with imaging reports and recorded examinations whenever possible.
Rehabilitation initiation	Initiate swallowing rehabilitation during acute hospitalization using physiologically targeted, task-specific approaches; avoid prolonged strict non-oral feeding without concurrent structured swallowing therapy incorporating therapeutic oral intake when physiologically appropriate.

**Table 2:** Post-acute care post-stroke dysphagia best practice recommendations

Domain	Best practice recommendation
Reassessment planning	Establish explicit reassessment timelines at discharge; treat repeat swallowing evaluation as an expected component of recovery-oriented care rather than an optional or resource-dependent service; prioritize imaging-based reassessment as neurologic recovery occurs, regardless of discharge disposition.
Swallowing imaging access	Pursue VFSS or FEES reassessment across post-acute settings to evaluate recovery, guide oral intake advancement, refine rehabilitation targets, and determine whether enteral feeding remains clinically necessary; address institutional and logistical barriers that limit swallowing imaging access in post-acute settings.
Enteral feeding re-evaluation	Reassess the continued need for enteral nutrition through repeat swallowing imaging as neurologic recovery progresses; establish structured pathways for gastrostomy tube removal when swallowing function has recovered sufficiently to permit safe and adequate oral intake; avoid indefinite enteral feeding without documented reassessment.
Continuity of care communication	Ensure that swallowing assessment findings, imaging results, oral intake recommendations, and rehabilitation goals are communicated consistently across care transitions between acute hospitals, inpatient rehabilitation facilities, skilled nursing facilities, outpatient services, and home health teams; minimize reliance on oral handoffs alone.
Swallowing rehabilitation	Provide access to skilled, physiologically informed dysphagia rehabilitation across the recovery continuum; use imaging findings to individualize rehabilitation targets; emphasize task-specific swallowing practice as a neuroplasticity-based mechanism supporting functional recovery.
Oral health	Integrate oral hygiene into routine post-acute and home-based care plans to reduce aspiration pneumonia risk; recognize that oral health is a modifiable determinant of pulmonary outcomes.
Nutrition integration	Incorporate systematic nutrition assessment into post-acute dysphagia management; monitor for persistent malnutrition or dehydration resulting from ongoing oral intake restrictions or enteral feeding dependence; engage dietitians as members of the interdisciplinary rehabilitation team.
Patient and care partner support	Provide anticipatory guidance reinforcing that swallowing recovery may continue beyond discharge; support patients and families managing home enteral feeding through structured training and access to follow-up resources; address psychosocial consequences of dysphagia including social isolation, anxiety, and caregiver burden.
Systems and reimbursement alignment	Advocate for reimbursement structures and care coordination mechanisms that support access to swallowing imaging, skilled dysphagia rehabilitation, and longitudinal reassessment across all post-acute care settings; use acute hospitalization documentation of swallowing impairment severity to establish medical necessity for post-discharge services.

*FEES* flexible endoscopic evaluation of swallowing, *VFSS* videofluoroscopic swallowing study

**Fig. 1:**
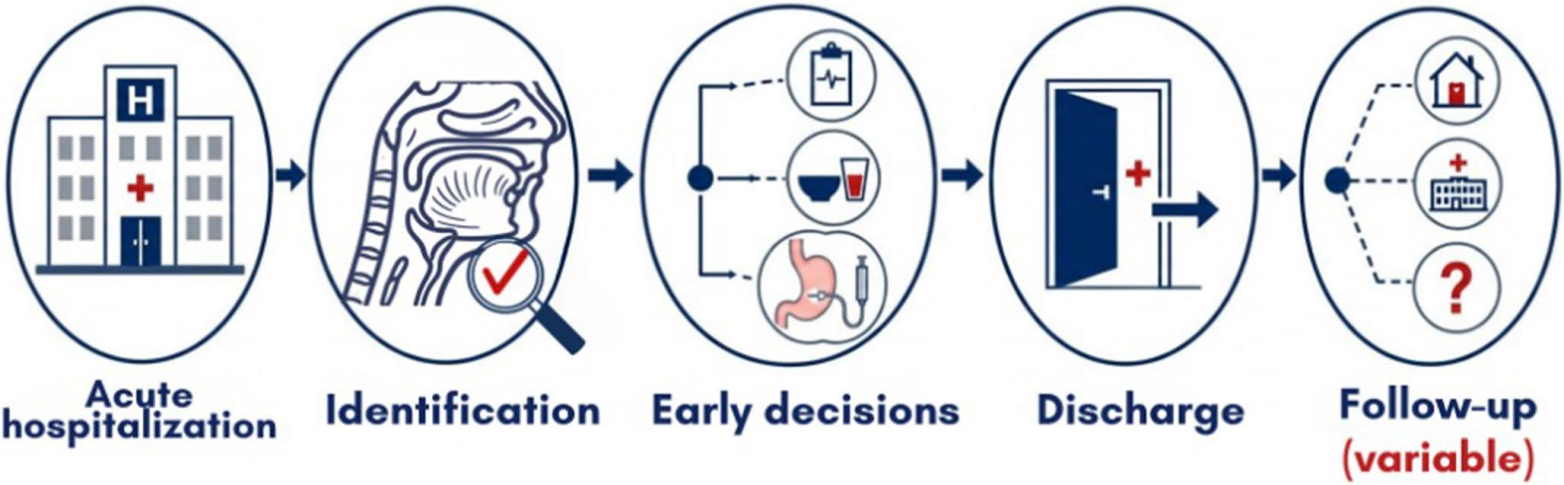
Simplified Care Pathway for Acute Care Post-Stroke Dysphagia. Dysphagia management begins during acute stroke hospitalization with identification of swallowing impairment and early clinical decisions regarding assessment, oral intake, rehabilitation, and nutritional support. Although these decisions are made during a brief hospitalization, they often influence long-term swallowing outcomes. Follow-up after discharge may be variable, underscoring the need for recovery-oriented care pathways that support reassessment, rehabilitation, and continuity of care across the stroke recovery continuum

Organized interdisciplinary stroke systems have consistently been associated with improved patient outcomes, underscoring the value of coordinated rehabilitation pathways and continuity across the recovery continuum [10]. Ultimately, improving outcomes after stroke requires recognizing that dysphagia management does not end at hospital discharge. Post-stroke dysphagia should not be viewed solely as a short-term inpatient safety issue, but rather as a dynamic rehabilitation condition requiring longitudinal management, structured reassessment, coordinated communication, and equitable access to rehabilitation services (Figure). What occurs during acute hospitalization profoundly influences the trajectory of recovery, with long-term outcomes ultimately dependent upon whether patients can access the reassessment, rehabilitation, and support necessary to translate recovery potential into meaningful functional improvement (Fig. 1).

## Limitations and Future Directions

Several limitations of the current evidence base warrant consideration. Much of the literature informing post-stroke dysphagia management derives from observational studies, registry analyses, retrospective cohorts, and expert consensus. Although these data consistently support the importance of dysphagia identification and management after stroke, high-quality prospective studies evaluating optimal timing of reassessment, swallowing imaging utilization, enteral feeding decisions, and post-acute care pathways remain limited. As a result, many aspects of contemporary dysphagia care continue to rely on extrapolation from heterogeneous populations and healthcare systems. Additional research is needed to better define recovery trajectories, identify patients most likely to benefit from specific rehabilitation approaches, and evaluate systems-level interventions designed to improve continuity of care. Future studies should also examine the effects of structured reassessment pathways, access to swallowing imaging across care settings, and patient-centered outcomes including quality of life, autonomy, and long-term feeding tube dependence.

## Conclusion

Post-stroke dysphagia remains an underrecognized determinant of stroke recovery and long-term health. Evidence increasingly supports recovery-oriented management including timely swallowing imaging, longitudinal reassessment, individualized rehabilitation, and coordinated transitions across care settings. Several priorities emerge from the current evidence. First, swallowing imaging should be recognized as a foundational component of post-stroke dysphagia management because accurate characterization of swallowing physiology is essential for diagnosis, rehabilitation planning, prognostication, and nutritional decision-making. Second, oral intake recommendations, restrictions, and decisions regarding enteral nutrition should account for the dynamic nature of swallowing recovery. Given the uncertainty of early prognostication, recovery-oriented care should avoid unnecessary long-term restrictions and support timely reassessment of patients with feeding tubes, including those who may ultimately recover sufficient swallowing function to permit G-tube removal. Third, systems of care must better support continuity across acute and post-acute settings through coordinated communication, structured reassessment pathways, patient and care partner education, and equitable access to rehabilitation and repeat swallow imaging. Many barriers to optimal outcomes reflect systems-level limitations rather than patient-level factors. A fundamental challenge in post-stroke dysphagia management is that many of the most consequential decisions regarding swallowing, nutrition, and rehabilitation are made during a hospitalization measured in days, whereas meaningful swallowing recovery often unfolds over months. Reframing post-stroke dysphagia as a rehabilitation condition requiring longitudinal management represents an important opportunity to better align stroke systems of care with the realities of recovery and improve long-term outcomes after stroke.

## Key References

- Hopkins-Rossabi T, Lenze A, Lindler SC, Hardy C, Temple SL. Analysis of patients' dietary status/restrictions following instrumental swallow evaluations in skilled nursing facilities. Dysphagia. 2025;40:476–488. https://doi.org/10.1007/s00455-024-10750-x.
  - o This study examined swallowing imaging findings in skilled nursing facilities and found that many patients receiving no oral intake and enteral feeding could safely resume some level of oral intake following reassessment. The findings support the importance of ongoing swallowing imaging and reassessment to prevent unnecessary long-term oral intake restrictions during stroke recovery.
- Karisik A, Moelgg K, Buergi L, Mayer-Suess L, Kneihsl M, Enzinger C, et al. Intensified post-stroke care improves long-term dysphagia recovery after acute ischemic stroke: results from the STROKE-CARD trial. Eur Stroke J. 2025;10(2):568–577. https://doi.org/10.1177/23969873241292802.
  - o This study found that stroke patients enrolled in a structured post-stroke follow-up program were significantly less likely to have persistent dysphagia at 12 months than patients receiving standard care. The findings support the importance of coordinated longitudinal care and reassessment in optimizing swallowing recovery after stroke.
- Braun R, Han K, Arata J, Gourab K, Hearn J, Gonzalez-Fernandez M. Establishing a clinical care pathway to expedite rehabilitation transitions for stroke patients with dysphagia and enteral feeding needs. Am J Phys Med Rehabil. 2024 May 1;103(5):390–394. https://doi.org/10.1097/PHM.0000000000002387.
  - o This study demonstrated the feasibility of a care pathway that facilitated discharge to rehabilitation with nasogastric tubes rather than early gastrostomy tube placement in selected stroke patients with dysphagia. The findings support the potential for coordinated care pathways to reduce unnecessary gastrostomy tube placement while allowing additional time for swallowing recovery and reassessment.

## Data Availability

No datasets were generated or analysed during the current study.
